# Exploring inflammatory signatures in arthritic joint biopsies with Spatial Transcriptomics

**DOI:** 10.1038/s41598-019-55441-y

**Published:** 2019-12-12

**Authors:** Konstantin Carlberg, Marina Korotkova, Ludvig Larsson, Anca I. Catrina, Patrik L. Ståhl, Vivianne Malmström

**Affiliations:** 1 Department of Gene Technology, Royal Institute of Technology, Science for Life Laboratory, Stockholm, Sweden; 2 Division of Rheumatology, Department of Medicine Solna, Karolinska Institutet, Karolinska University Hospital, Stockholm, Sweden

**Keywords:** Transcriptomics, Autoimmunity

## Abstract

Lately it has become possible to analyze transcriptomic profiles in tissue sections with retained cellular context. We aimed to explore synovial biopsies from rheumatoid arthritis (RA) and spondyloarthritis (SpA) patients, using Spatial Transcriptomics (ST) as a proof of principle approach for unbiased mRNA studies at the site of inflammation in these chronic inflammatory diseases. Synovial tissue biopsies from affected joints were studied with ST. The transcriptome data was subjected to differential gene expression analysis (DEA), pathway analysis, immune cell type identification using Xcell analysis and validation with immunohistochemistry (IHC). The ST technology allows selective analyses on areas of interest, thus we analyzed morphologically distinct areas of mononuclear cell infiltrates. The top differentially expressed genes revealed an adaptive immune response profile and T-B cell interactions in RA, while in SpA, the profiles implicate functions associated with tissue repair. With spatially resolved gene expression data, overlaid on high-resolution histological images, we digitally portrayed pre-selected cell types *in silico*. The RA displayed an overrepresentation of central memory T cells, while in SpA effector memory T cells were most prominent. Consequently, ST allows for deeper understanding of cellular mechanisms and diversity in tissues from chronic inflammatory diseases.

## Introduction

Rheumatoid arthritis (RA) and spondyloarthritis (SpA) are common chronic inflammatory diseases characterized by joint inflammation^1^. Inflamed joints in both disorders are characterized by infiltrating leukocytes as well as local proliferation of mesenchymal cells, hence affected joints from both disorders display a multitude of inflammatory cells and mediators. Joint biopsies from both RA and SpA have been extensively studied and compared^2^. The shared features include T cell infiltrates and biomarkers such as the cytokine TNF, which can be readily visualized by classical immunohistochemistry^3,4^.

Clinically, RA and SpA are regarded as complex heterogeneous autoimmune diseases. Although the symptoms and treatments of these two diseases are partly similar, RA and SpA do not share etiology and their pathophysiological mechanisms differ^5,6^. The genetic predisposition for RA has been linked to variants in the MHC class II genes (the so-called shared epitope HLA-DR alleles)^7,8^, while SpA is linked more closely to the MHC class I pathway with HLA-B27 and the proteasome components encoded by *ARTS-1* as the major disease predisposing genes^6^.

In recent years, it has become increasingly apparent that RA can, and at least for research purposes should, be divided into broad subsets, with seropositive RA as the major subset representing a classical autoimmune disease as defined by HLA class II association and autoantibodies^9–12^. The genetic associations in seropositive RA implicate adaptive immune responses in the disease pathogenesis and data on isolated cell subsets have pinpointed the CD4+ T cells as central players^13,14^. Spondyloarthritis on the other hand, is not characterized by autoantibodies and the predominance of myeloid alterations suggests that SpA rather represents autoinflammatory disease processes, with stronger association to mechanisms of innate immune responses^15^.

Our current understanding of the inflammatory pathways is largely based on studies of synovial fluid and synovial biopsy material, which both display a high degree of heterogeneity of cell composition. The use of biopsies for transcriptomic approaches has therefore been hampered by the low interpretability of gene profile data, resulting in classical immunohistochemistry (IHC) still being the method of choice in biopsy studies. However, in recent years the field of transcriptomics has evolved and become a more common way to study the regulatory molecular pathways of cells even with mixed composition. Transcriptomic profiling of biopsy material and single-cells in peripheral blood and from biopsies have successfully revealed impacts of drug treatments, disease activity and distinct pathogenic processes^16–21^. These approaches, although informative have mostly focused on cells from homogenized tissues or single cell suspensions whereby the spatial proximity and cellular context is lost.

In this study we have explored RA and SpA synovial tissues using the Spatial Transcriptomics (ST) technology, which combines histological imaging and RNA-Seq by retaining the positional information for each transcript through spatially immobilized and barcoded cDNA synthesis primers^22^. The spatially resolved mRNA data allows us to focus on specific tissue regions, in our case where infiltrating leukocytes organize into cell dense areas, i.e. infiltrate regions, and areas in between infiltrates. This enables for orientation in the complex microenvironment of the inflamed tissue, in order to find novel gene expression characteristics of RA and SpA in distinct locations.

## Methods

### Patients and samples

Three patients with seropositive RA (ACPA-positive and/or RF positive, HLA-DR shared epitope-positive) and three patients with SpA were included in the study. Clinical data of patients are presented in Supplementary Fig. S1A,B.

The synovial tissue biopsies from knee or hip joints were collected during orthopedic total replacement surgery. The approval was granted by the Ethic’s Committee at the Karolinska University Hospital, Stockholm and all patients gave their informed consent to participate in the study. All experiments were performed in accordance with the relevant guidelines and regulations. The tissue samples were snap frozen in isopentane prechilled with liquid nitrogen and stored at −70 °C until sectioning.

### Spatial transcriptomics

The Spatial Transcriptomics protocol was carried out as previously described.^22,23^ Tissue permeabilization and tissue removal parameters were optimized for synovial tissue (Supplementary Figs. S1 and S2). The Hematoxylin and Eosin (H&E) stained tissue sections images were annotated for mononuclear cell infiltrates (Supplementary Fig. S3). The selection criteria were based on biopsy size (covering >100 spots), data depth (>80,000 transcripts for the whole tissue), and morphology with presence of infiltrates and little to no damage.

Three near adjacent sections were selected for each patient. The protocol was prepared with some minor differences. The surface probe release step was carried out for 3 h at 37 °C. Final libraries were purified and validated using an Agilent Bioanalyzer (with the DNA 1000 or DNA HS kit) and Qubit before sequencing on the NextSeq. 500 (v2) at a depth of ~60–100 M reads per tissue section. The forward read contained 31 bases and the reverse read 46 bases.

### Data processing and image annotation

Data processing was carried out as previously described^22,24^. The analysis pipeline used (v0.8.5) is available at https://github.com/SpatialTranscriptomicsResearch/st_pipeline.

Briefly, mapping was performed to the reference GRCh38 human genome. The demultiplexed reads were then filtered for amplification duplicates using the UMI with a minimal hamming distance of 2. The UMI-filtered counts were used in the analysis. Ambiguous counts were filtered out for the analysis, as well as pseudogenes, lncRNA by mapping to only coding mRNAs. A list of RPS and RPL genes were also filtered out since these genes turned out to have extreme variance between the samples (https://www.spatialresearch.org/resources-published-datasets/).

The count matrixes used for the analysis can be found in https://www.spatialresearch.org/resources-published-datasets/.

The image alignment and processing were conducted as previously described. A detailed manual of the image processing is available at https://www.spatialresearch.org/resources-computational-tools/spatial-transcriptomics-spot-detector/.

### Data analysis

Briefly, data analysis was carried out through steps of normalization and clustering, followed by differential gene expression analysis (DEA) using edgeR and pathway analysis using Metascape and Ingenuity Pathway Analysis (IPA)^25–27^. Lastly, cell types were also predicted and visualized using Xcell analysis^28^.

### Data filtering for analysis

Ensembl gene symbols were converted to RefSeq-ids to ease processing. All genes with total counts over all spots within one section with at least 10 counts and present in at least two spots were kept for analysis.

### Normalization and clustering

The data was normalized with counts per 10,000 for each spot and log2 converted. Thereafter, clustering was performed using hierarchical clustering. The method was the number of clusters generated with a dynamic tree cut^25^. Each section was clustered individually due to the morphological differences between them.

In order to utilize all generated data and obtain an average gene expression pattern over all morphological landscapes, the gene counts were summarized for each tissue section. The data from all tissue sections (three sections per patient) was then normalized with the Counts per Million (CPM) approach and subjected to Differential expression analysis (DEA). The differentially expressed genes were identified with an empirical Bayes method followed by a quasi-likelihood F-test and likelihood ratio test providing adjusted *p*-values, using the Bioconductor package edgeR^26^.

### Annotation and selection of clusters

Specific clusters were selected based on the morphological areas assigned as infiltrate regions, as well as regions outside of infiltrates, which the clusters were matched to (Supplementary Fig. S3). The infiltrate comparison clusters that were strongest correlated to the annotated regions were used. For clusters with non-obvious overlap, the clusters with most spots over the infiltrates were selected. In this case more data points could be used to strengthen the statistical power. In cases where two clusters were correlated to infiltrates both were merged and used for the analysis. For the clusters outside infiltrates all fibrotic tissue areas were excluded. All raw H&E images used for this study with histological annotation for infiltrates and generated clusters can be found in https://www.spatialresearch.org/resources-published-datasets/.

### Differential expression analysis and pathway analysis

For the Differential Expression Analysis (DEA) all gene counts for each individual tissue section were summed and normalized with CPM and log2 converted. The DEA was performed on the conditions RA and SpA using an empirical Bayes method followed by a quasi-likelihood F-test and likelihood ratio test providing adjusted *p*-values, within the Bioconductor package edgeR^26^. The DEGs generated were subjected to a functional analysis of the molecular networks and pathways using Ingenuity Pathway Analysis (IPA; Ingenuity Systems Inc, www.ingenuity.com) and Metascape (http://metascape.org).

### Visualization of cell types with xCell analysis

The recently published xCell method was used in order to assign and visualize immune cell types onto tissue sections^28^. Firstly; the data was normalized with counts per 10,000 for each feature and log2 converted. The log2 normalized counts were subjected to a linear discriminant analysis from the dynamic tree dynamicTreeCut package in R^25^. This analysis was performed pairwise with two sections at a time. Thereafter the samples were clustered using lda modeling from the cellTree package with the maptpx method. The HE-images were converted to scatter images and thereafter rasterized and the data from the clusters were interpolated. The interpolated data were thereafter visualized with xCell with enrichment scores for the selected cell types. This score for each cell type on the heatmap is explicitly showing the enrichment of the certain cell type compared to other regions within the same section. Therefore, xCell only works as a visualization tool and not a quantitative analysis. Such visualization would, if performed by classical immunohistochemistry, require a multi-color approach.

### Immunohistochemical analysis

The tissue samples were snapfrozen in isopentane prechilled with liquid nitrogen and stored at −70 °C until sectioning. Cryostat sections (7 µm) were fixed with 2% formaldehyde (Sigma) for 20 minutes. Sections were incubated with monoclonal mouse α-CD3 (BD Biosciences) or polyclonal goat α-CD138 (R&D systems) antibodies according to previously described protocol^29^. Isotype matched irrelevant antibodies were used as negative control. The staining sections were examined using a Polyvar II microscope (Reichert-Jung, Vienna, Austria) and Leica Qwin IM500 software (Leica, Cambridge, UK).

### Ethics approval

For this study informed consent from the patients have been obtained and recognized by the regional ethics committee in Stockholm. This study was approved 2009-09-09 with registration number Dnr: 2009/1262-31/3.

## Results

### Differentially expressed genes in RA and SpA joint tissue

We first optimized the RNA capture step, including tissue permeabilization conditions for synovial tissue (Supplementary Fig. S1A–C). Thereafter we applied the ST technology on the synovial tissue sections (Supplementary Fig. S1C).

We obtained positional information for approximately 9 million unique transcripts captured across a total of 6,240 spatially barcoded spots. In two out of three cases the RA sections were larger than the SpA samples. The average number of uniquely mapped genes per tissue section was higher for RA than for SpA (14500 ± 815 and 12300 ± 1550 respectively (https://www.spatialresearch.org/resources-published-datasets/).

**Figure 1:**
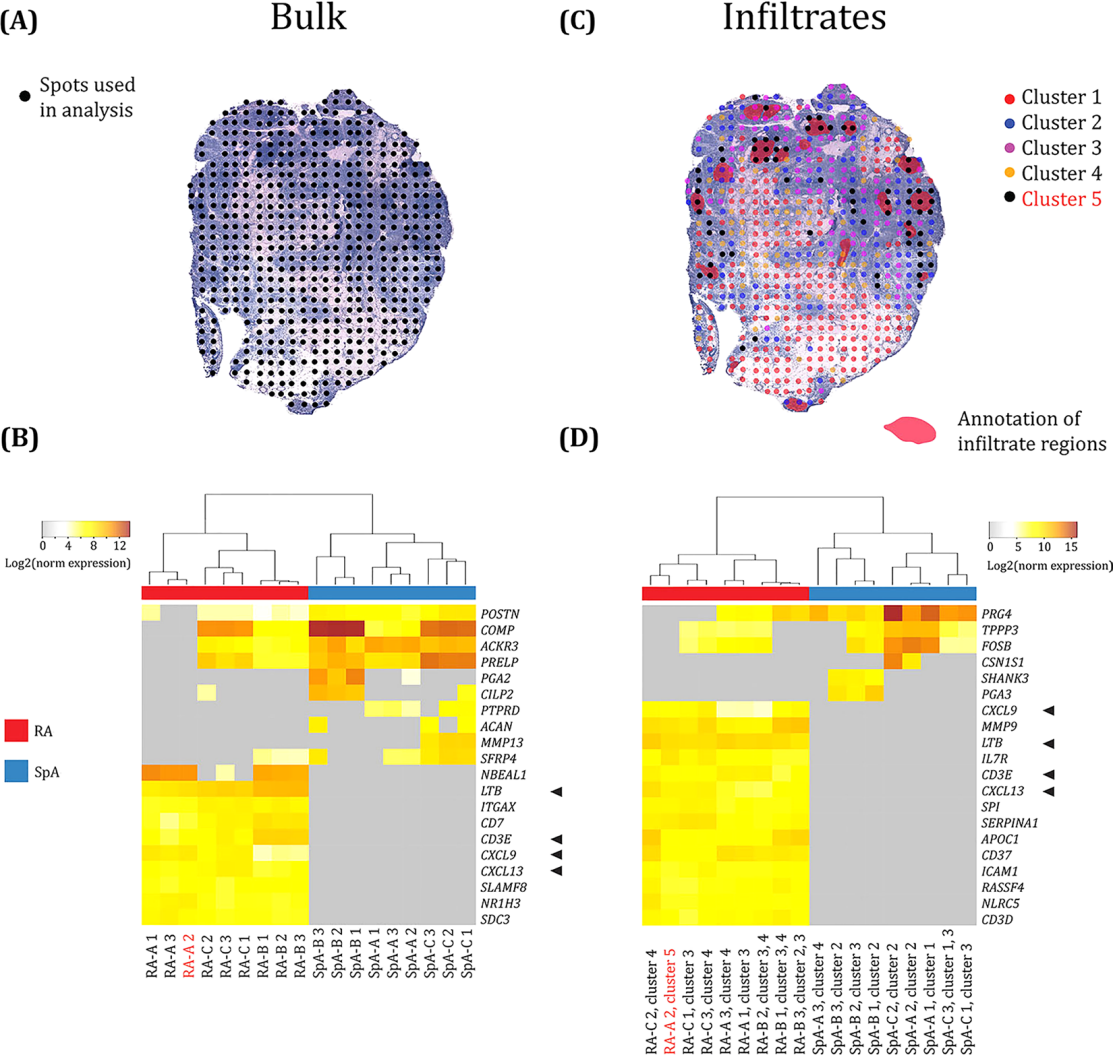
Comparison of the top differentially expressed genes in RA vs. SpA when analysing the entire tissue section as a bulk compared to pre-selection of morphologically interesting areas. **(A)** An example of an H&E stained RA tissue section and the spots were the local transcriptome was captured in black. **(B)** A heatmap with the top 20 differentially expressed genes (DEGs) in the bulk data (without pre-selecting cluster) and ordered by lowest *p*-values (from top). The colour labeling represent the log2 normalized expression values and only genes with greater than twofold the difference in normalized expression value were selected. Each column represents a section, and three sections per patient were used. All sections were treated as a bulk with red columns representing RA and blue representing SpA. The tissue sections from the same biopsies cluster together and the SpA samples are clearly different to the RA samples. **(C)** The same tissue section with the annotated lymphocyte infiltrate areas in red and the unbiased analysis showing which spots fall into different clusters (depicted by different colours) as generated with an hierarchical clustering approach. For this tissue section, the black cluster (cluster 5) represents the cluster with strongest association to the histological annotation (red areas). **(D**) A heatmap with the top 20 differentially expressed genes (DEGs) in the pre-selected clusters overlapping the annotated infiltrates and ordered by the lowest *p*-values (from top). The colour labeling represent the log2 normalized expression values and only genes with greater than twofold the difference in normalized expression value were selected. Each column represents a selected cluster from each tissue section, and three sections per patient were used with red columns representing RA sections and blue representing SpA. The arrowheads show differentially expressed genes seen in both the bulk analysis and the infiltrates analysis.

In the first analysis the spatial data from each section was pooled into a bulk dataset including data from all tissue sections (three from each patient) (Fig. 1A). Here, we found 796 differentially expressed genes in RA versus SpA using a cutoff of log2fold-change > 2 and *p*-value < 0.01 (https://www.spatialresearch.org/resources-published-datasets/ and Supplementary Fig. S4). The top 20 differentially expressed genes (DEGs), sorted by *p*-value, include upregulated genes involved in adaptive immune responses in the RA tissue, while in the SpA tissue, the genes encoding more general immune mediators were upregulated (Fig. 1B).

Overall, both diseases showed distinct molecular signatures where RA showed stronger associations with T cells, as well as TNF, while the SpA tissues were more characterized by processes related to cartilage damage and repair systems. The data further supports evidence for Ectopic Lymphoid Structures (ELS) in RA and a more hyper- and dysplastic gene expression profile for SpA (Fig. 1B).

### Differential gene expression profiles in the mononuclear cell infiltrates

Next, the spatial gene expression data from each individual tissue section was subjected to hierarchical clustering. In parallel, histological annotation on mononuclear cell infiltrates was carried out based on the H&E-staining as shown in Fig. 1C. In order to study molecular signatures, we selected the gene clusters that associated with the infiltrate regions. We hypothesized that a comparison of the clusters over similar morphological regions between RA and SpA would provide a higher resolution profile of key differentially expressed genes driving the respective inflammatory states. Using this approach, we found 660 differentially expressed genes associated with infiltrate areas (https://www.spatialresearch.org/resources-published-datasets/ and Supplementary Fig. S5). The 20 top differentially expressed genes were visualized in a heat map (Fig. 1D), and four of these, *CXCL9, LTB, CXCL13* and *CD3E*, were also seen in the bulk analyses, and were hence both globally and locally overexpressed in the RA tissue as compared to SpA. Many of the overexpressed genes in the RA mononuclear aggregates including *CXCL13*, *LTB, SP1* and *CD37* are known to be associated with germinal centers and ectopic lymphoid structures and these genes were absent in SpA (Fig. 1D)^30,31^. For SpA, a higher expression of *PRG4*, which is produced by synovial fibroblasts and superficial zone chondrocytes, and also seems to be involved in the regulation of synoviocyte proliferation, as well as genes encoding catabolic and cartilage degradation markers could be seen^32^. Further, the upregulation of the genes *TPPP3* and *FOSB* indicates ongoing cell proliferation, differentiation, and transformation. Of all the DEGs with the cutoff of log2fold-change >2 and *p*-value < 0.01, 187 were seen both in the bulk analysis and the infiltrate analysis.

We also studied gene clusters, associated with areas outside the infiltrate regions, excluding acellular fibrotic areas (Supplementary Fig. S6). These regions were subjected to differential gene expression analysis (DEA) as previously described. We observe different profiles between RA and SpA also in these areas. However, the genes were not identical but still associated with similar functions as compared to the infiltrate analysis.

### Pathway analysis of differentially expressed genes

**Figure 2:**
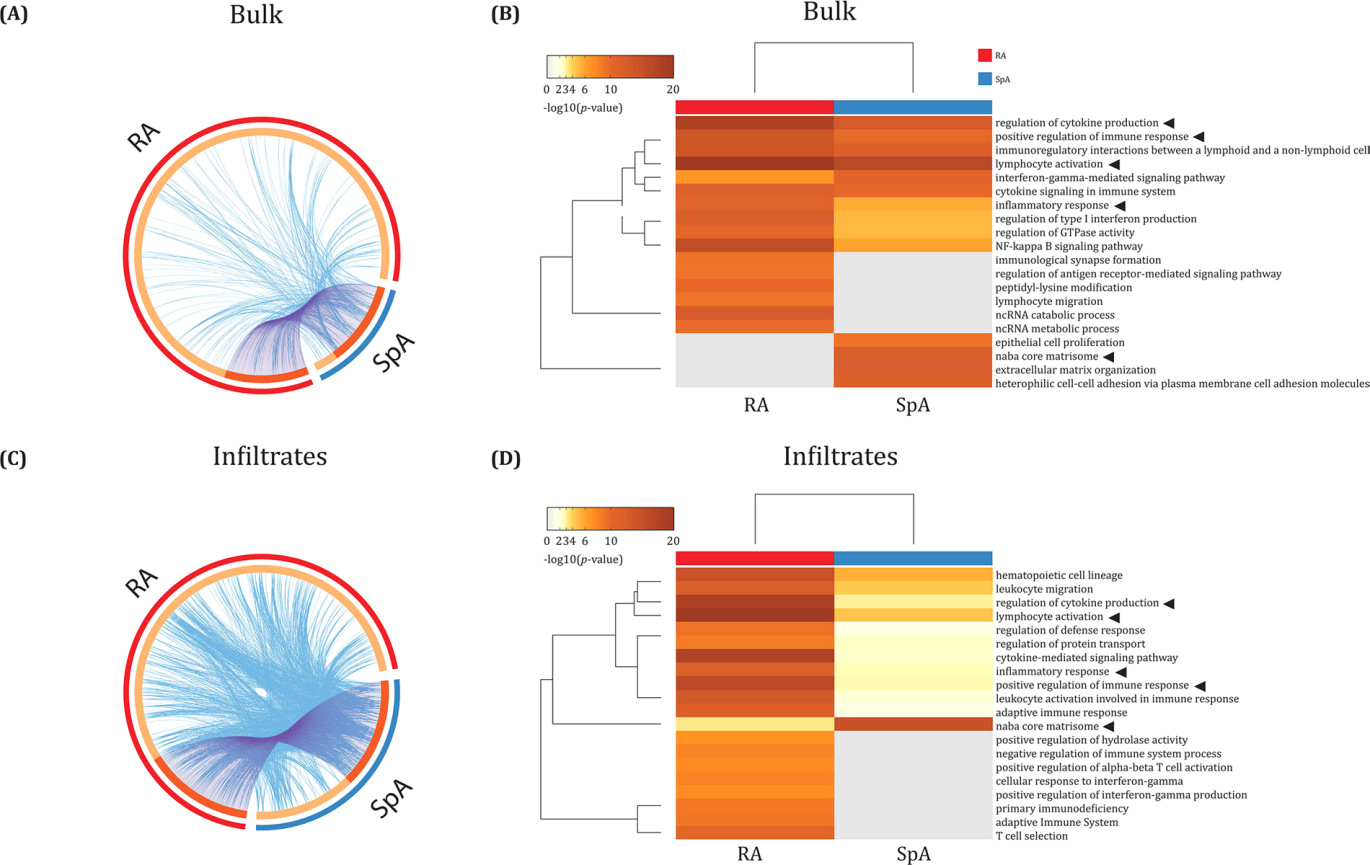
Shared and unique pathways prominent in affected joint tissue from RA versus SpA. **(A)** Circos plot showing overlapping genes from the *in silico* bulk analysis based on differentially expressed genes. Each segment of the inner circle represents a gene list, whereas each line is originating from a single gene. Segments colored dark orange cover genes appearing in both the RA and SpA gene lists while light orange represents unique genes. Purple lines connect the genes being shared in the RA and SpA gene lists. Blue lines link the different genes where they fall into the same ontology term. A high number of connecting purple lines as well as a long dark orange segment thus implies an extensive overlap in the RA and SpA gene lists. **(B)** Heatmap showing the top 20 pathways from metascape analysis based on differentially expressed genes, sorted by *p*-value. **(C)** Circos plot of selected clusters associated with infiltrates shows that the higher resolution results in a stronger similarity between the infiltrate genes in diseases as well as higher heterogeneity within the diseases. **(D)** Heatmap showing the top 20 pathways for the analysis of selected clusters. Cluster selection was based on the association with infiltrates. The higher resolution obtained by selecting clusters reveals different pathways than the bulk analysis. The arrowheads show pathways overlapping both the bulk analysis and the infiltrates analysis.

In the bulk analysis 100 differentially expressed genes were present in both RA and SpA (although at significantly different levels), whereas 670 genes were expressed only in RA and 26 genes only in SpA. In addition to the 100 shared genes, there were a number of genes with functional overlap across the RA and SpA gene lists (Fig. 2A). Gene ontology (GO) analysis further demonstrated that the shared genes from the RA and SpA bulk analysis were connected to biological processes related to immune functions (Positive regulation of immune response, Lymphocyte activation, Immunoregulatory interactions between a lymphoid and a non-lymphoid cell, and Regulation of cytokine production) (Fig. 2B). In addition, more terms related to adaptive immune response, such as Immunological synapse formation, Lymphocyte migration and Regulation of antigen receptor-mediated signaling pathway were found to be significantly enriched in the RA data set. In contrast, the SpA data set was enriched for such terms as Extracellular matrix organization, Naba core matrisome and Heterophilic cell-cell adhesion via plasma membrane cell adhesion molecules, reflecting processes related to extracellular matrix.

More specifically, the IPA analysis for bulk data demonstrated that Th1 and Th2 activation pathways, as well as TWEAK signaling were prominent in both diseases (Supplementary Table S1). In RA, the unique top canonical pathways included iCOS-iCOSL signaling in T helper cells, Regulation of IL-2 expression in activated and anergic T lymphocytes, CD28 signaling in T helper cells, T cell receptor signaling and PKCθ signaling in T lymphocytes, reflecting the crucial role of T cells in the RA pathogenesis. In SpA, various canonical pathways were identified such as Tumoricidal function of hepatic natural killer cells, Role of osteoblasts, osteoclasts and chondrocytes in RA, Atherosclerosis signaling, Basal cell carcinoma signaling, Calveolar-mediated endocytosis signaling and Glutathione biosynthesis, indicating also involvement of non-immune pathogenic mechanisms in SpA. Difference in the molecular mechanisms in RA and SpA was also confirmed by the analysis of upstream regulators. In RA, the top upstream regulators included IL-12, IL-21, IL-2, SERPINE2 and CD40LG while in SpA the top upstream regulators were: TGFB1, IFNG, MAP3K14, SIRT1 and Alpha catenin (Supplementary Table S2, Supplementary Fig. S7A).

In the infiltrate analysis 112 differentially expressed genes were present in both RA and SpA (although at significantly different levels), whereas 445 genes were expressed only in RA and 103 genes only in SpA. In addition to the 112 shared genes, there were a number of genes with functional overlap across the RA and SpA gene lists (Fig. 2C). The functional overlap between RA and SpA infiltrates was greater than between the RA and SpA bulk gene lists. Gene ontology (GO) analysis further demonstrated that the RA and SpA shared genes from the infiltrate areas were connected to biological processes such as lymphocyte activation, leukocyte migration and regulation of cytokine production (Fig. 2D). In RA infiltrate areas, the subgroups of genes with terms related to adaptive immune system such as T cell selection, Cellular response to interferon gamma, Positive regulation of alpha-beta T cell activation and Positive regulation of INFG production were enriched. In the SpA infiltrate data set an enrichment of genes associated with Naba core matrisome was identified, similarly to the results obtained in the bulk analysis.

Furthermore, pathway analysis using IPA demonstrated that CD28 signaling in T helper cells and iCOS-iCOSL signaling in T helper cells pathways were prominent in both diseases in infiltrate areas (Supplementary Table S3). In RA, the unique top canonical pathways included Regulation of IL-2 expression in activated and anergic T lymphocytes, Th1 and Th2 activation pathways, T cell receptor signaling, PKCθ signaling in T lymphocytes, Calcium-induced T lymphocyte apoptosis and OX40 signaling pathways reflecting pathogenic role of T cell response. In SpA infiltrate areas, the following unique canonical pathways were present such as Role of osteoblasts, osteoclasts and chondrocytes in RA, Axonal guidance signaling, Virus entry via endocytic pathways, Leukocyte extravasation signaling, CDC42 signaling, CCR5 signaling in macrophages, Role of NFAT in regulation of the immune response and Hepatic fibrosis/hepatic stellate cell activation, again demonstrating significance of non-immune pathogenic mechanisms in SpA even in infiltrate areas.

Moreover, among top upstream regulators, IFNG was found both in RA and SpA (Supplementary Table S4, Supplementary Fig. S7B). In RA infiltrates, other upstream regulators were IL-2, CD3, NFKB1 and CD40LG. Unique upstream regulators in SpA infiltrates were TGFB1, CHUK, NOTCH1, IRS1, SIRT1 and GATA.

Pathway analysis using IPA and Metascape was also conducted on the regions outside infiltrates, see Supplementary Tables S5 and S6 and Supplementary Fig. S8.

### Cell type assignment using xCell

As a further means of visualization of tissue heterogeneity, and to predict immune cell patterns in the samples we utilized the xCell^28^ algorithm, which shows the relative enrichment of predetermined combinations of gene profiles. We selected six different immune cell signatures central for adaptive immune responses including both central and effector memory T cells of both the CD4 and CD8 lineages as well as class switched B cells and plasma cells. Due to the highly heterogeneous nature of the tissue sections from the different patients, the xCell analysis was conducted on one of the samples of each disease, both having similar morphology with comparable lymphocyte aggregates based on the number of spots covered by infiltrates (106 RA-B 3 and 109 in SpA-A 1).

**Figure 3:**
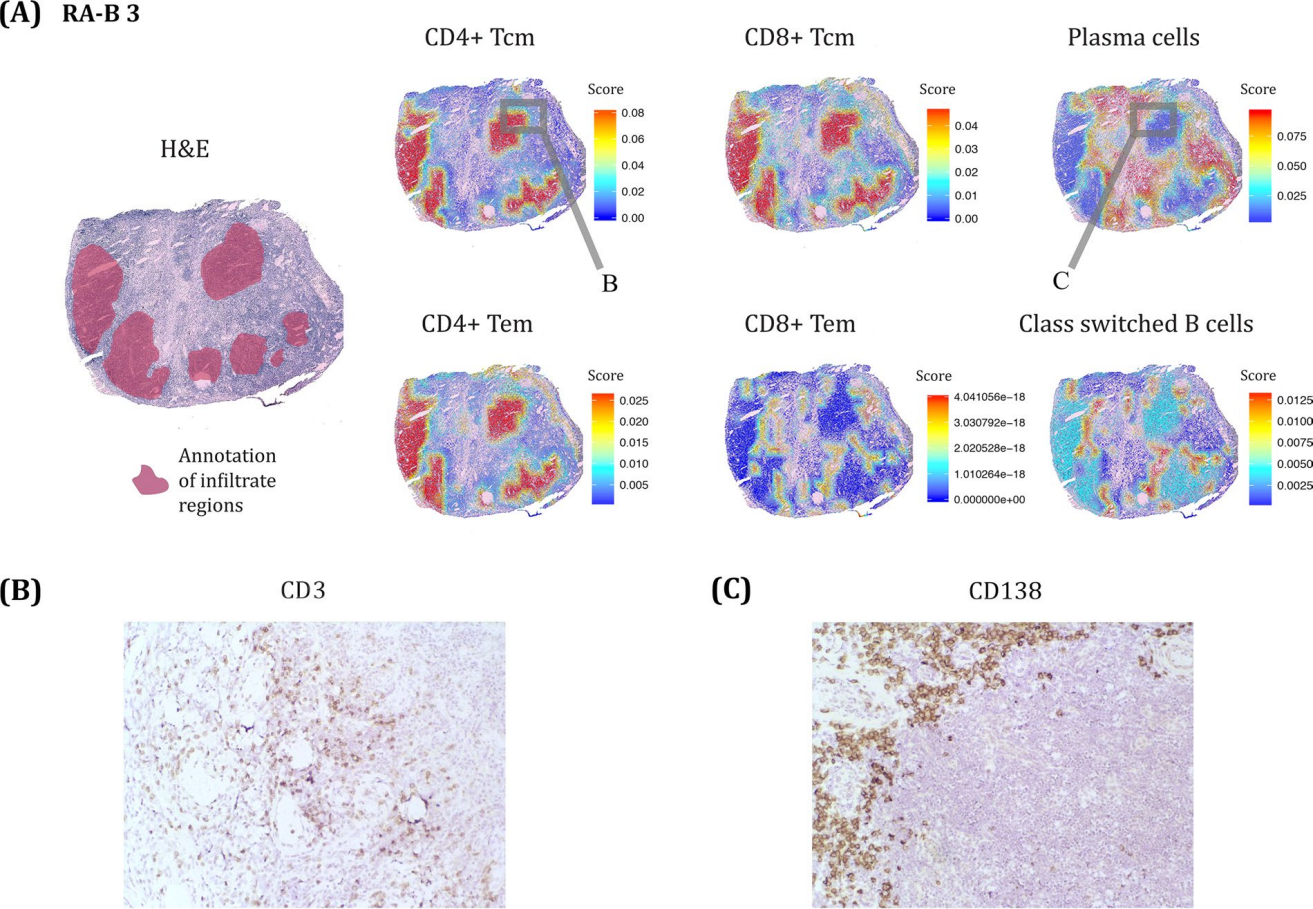
The RA lymphocyte infiltrates are enriched in central memory CD4 and CD8 T cells as well as CD4 effector memory T cells. **(A)** Examples from one of the RA tissue sections depicting both the H&E stained section with the annotated mononuclear infiltrates as well as visualizations of transcriptomic data to show the localizations of different lymphocyte subsets as assigned by xCell (v1.1.0) method and viewed as spatial heatmaps to the right. The value for the respective cell subsets is an enrichment score showing how much enriched each cell type is compared to other regions within each individual tissue section. N.B. it is not possible to compare the amount of cell types for the sections. For that reason significance numbers were not considered for this figure. (**B**) Validation of xCell data with IHC analysis of T-cells (CD3-positive) from a nearby section in an area corresponding to the grey box on the CD4+ Tcm image in **(A)**. **(C)** Validation of xCell data with IHC analysis of plasma cells (CD138-positive) from a nearby section in an area corresponding to the grey box on the plasma cell image in **(A)** with expected reciprocal T cell and Plasma cell distributions.

For the RA sample, the infiltrate regions included T cells of both the CD4 and CD8 T cell lineages. Regarding the B cells, plasma cells were found to reside outside of the annotated infiltrate regions while the switched B cells were found in the border regions of the aggregates. These results are in line with previously known data from the many studies of RA tissue with immunohistochemistry or immunofluorescence approaches^33^. When looking into the relative distribution of the effector and central memory T cell subsets (Tem and Tcm respectively), both CD4 subsets were prominent and largely overlapping, while the CD8 subsets were reciprocal in their spatial localization. Indeed, the CD8 Tem was the T cell subset that most matched the position of the switched B cells (Fig. 3). In order to validate the xCell distribution we used IHC to stain for CD3+ T cells and CD138+ plasma cells respectively in sections from the same RA biopsy as depicted in Fig. 3B,C. As can be seen, our protein stainings and the RNA signatures are in agreement.

**Figure 4:**
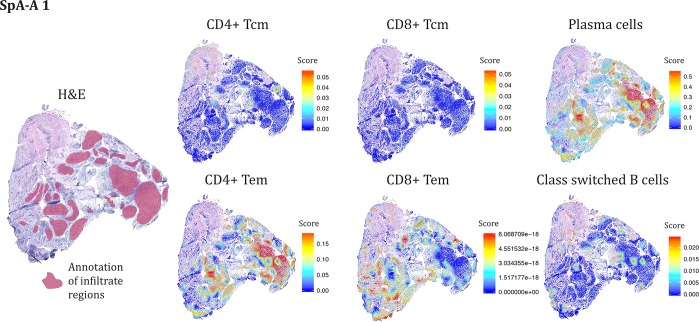
The SpA lymphocyte infiltrates are enriched in CD4 effector memory T cells and lack signals for the central memory T cells. Examples from one of the SpA tissue sections depicting both the H&E stained section with the annotated lymphocyte infiltrates as well as visualizations of transcriptomic data to show the localization of different lymphocyte subsets as assigned by xCell (v1.1.0) method and viewed as spatial heatmaps to the right. The value for the respective cell subsets is an enrichment score showing how much enriched each cell type is compared to other regions within each individual tissue section. N.B. it is not possible to compare the amount of cell types for the sections. For that reason significance numbers were not considered for this figure.

For SpA sample, the central memory T cells of both the CD4 and CD8 lineage were relatively absent from the annotated lymphocyte regions, while clear signals for the effector memory subsets were seen, albeit with only partial spatial overlap (Fig. 4). And again, the switched B cells and plasma cells distributed in a reciprocal fashion in the tissue.

Thus, our morphological annotation represented the T cell-rich areas of the tissue well, and the xCell analysis provides an important approach for guiding the selection of regions of interest for deeper analysis, based on signatures associated with lymphocytes with different levels of differentiation.

## Discussion

We have performed a comprehensive study of the transcriptomic profiles of RA and SpA synovial tissue samples by utilizing a new technology for spatially resolved gene expression analysis across intact tissue sections. With the retained spatial information we could characterize disease specific gene expression in a higher resolution than previously possible. By combining the spatial gene expression with high-resolution morphological imaging we can compare the data from regions of interest in an unbiased way. In our differential gene expression analysis and pathway analysis, on both tissue section and lymphocyte infiltrate levels, we have identified key genetic components that seem to differentiate pathogenic mechanisms in RA and SpA.

From the RA tissue analyses we could validate and extend the central role of adaptive immune responses and local germinal center reactions in affected peripheral joints. Interestingly, four out of the top 20 DEGs from the mononuclear cell infiltrates in RA synovia were also among the top 20 DEGs when analyzing the whole tissue sections without the spatial focus. These were *CD3E*, *CXCL9*, *CXCL13* and *LTB* which all fall into T cell function. CXCL9 is a ligand for the Th1 associated CXCR3 chemokine receptor, which is widely expressed on T cells in the RA joint and also on citrulline-reactive T cells in RA patients^34^. CXCL13 on the other hand is a ligand for CXCR5, which is found on so called follicular helper T cells, i.e. T cells in the germinal center. CXCL13 is also produced by the recently described peripheral helper T cells revealed in the RA joint^35^. Interestingly, *LTB* has been demonstrated to be required for the formation of lymph nodes^36^, and as such could be viewed to contribute in the ectopic germinal center formations seen in RA patients, structures that have been suggested to be present in SpA as well^30,31,37^.

From the SpA tissue analyses we suggest that the upregulated expression of *POSTN*, *COMP*, *CILP2* and *PRG4* associates with the higher cartilage turnover in SpA compared to RA^32^. The SpA tissue expressed more mesenchymal cell signatures, even when the analysis was focused on mononuclear infiltrates, which resulted in a profile similar to fibrotic tissue. This fits with the notion of matrix and bone regeneration, which is characteristic of SpA.

Still, using the xCell visualization tool, we observe that T cell and B cell signatures were prominent in both diseases, and largely overlapping with the infiltrate regions. Also, we could observe T cell signatures also outside of the lymphocyte aggregates and these were primarily effector memory T cells of both lineages. It would be interesting to build additional gene profile signatures, e.g. for the peripheral helper T cells described in ACPA+ RA joint^35^ in order to confirm their absence in SpA tissue. Moreover, there are several interesting B cell subsets that could be studied as well, such as the age-associated B cell subset^38^. The use of this visualization tool would also be an interesting alternative for the selection of tissue subregion to focus on in the Spatial Transcriptomics data set.

Although we have generated comprehensive transcriptomic profiles for inflamed RA and SpA synovial tissues and successfully performed immune cell predictions onto tissue sections, we consider this to be an exploratory study. We acknowledge the limitation of only having few study subjects with biopsies of varying morphology. This makes it challenging to draw conclusions only based on gene expression profiles. Still, several of the pathways we find would merit further studies, e.g. the naba core matrisome in SpA.

Spatial Transcriptomics provides a straight-forward way to obtain molecular profiles at high throughput, yet the resolution is currently limited. Higher resolution, when applied to our samples, would open up the possibility to study for example clonality, cell migration pathways, cell-to-cell interactions and cross talk. We also open up the possibility to visualize consecutive adjacent tissue sections to build 3D models of gene expression patterns across the infiltrate areas. This analysis could be further supplemented with trajectory analysis, showing pseudotime paths for each spot, which potentially could provide insights on the direction of cell activation or migration.

## Supplementary information

Supplementary materials

## Data Availability

The raw sequencing datasets generated and analysed during the current study are available in the PRJNA580481 repository, https://www.ncbi.nlm.nih.gov/bioproject/580481. High-resolution H&E images and count matrices are available in https://www.spatialresearch.org/resources-published-datasets/.
