# Supplementary material for: Exploring inflammatory signatures in arthritic joint biopsies with Spatial Transcriptomics
Source: Sci Rep. 2019 Dec 12;9:18975. doi: 10.1038/s41598-019-55441-y (PMC6908624; doi:10.1038/s41598-019-55441-y)
Supplement: Supplementary file 1 — Supplementary materials [file 41598_2019_55441_MOESM1_ESM.pdf]

Supplementary materials

Exploring inflammatory signatures in arthritic joint biopsies with Spatial Transcriptomics

Konstantin Carlberg, Marina Korotkova, Ludvig Larsson, Anca I Catrina, Patrik L Ståhl, Vivianne Malmström

Supplementary methods

Study design

An equal number of patients was selected for each disease (n=3), Fig. S1A-B. The samples were prepared according to the ST protocol as previously described (Fig. S1 C).[24]

RNA quality control

Before the method optimization, the RNA quality was analyzed by extracting total RNA from the biopsies, as previously described.[24] Ten 10µm sections of each biopsy were used for determining the RNA Integrity Number (RIN) which was between 4-9.2 for all samples.

Method optimization for RA and SpA tissue for Spatial Transcriptomics

The optimal conditions for tissue permeabilization and tissue removal for the Spatial Transcriptomics protocol were determined. This process uses cDNA synthesis with Cy3-labeled dCTPs to create a fluorescent footprint of cDNA, as previously described.[1] For RA and SpA tissues, the following parameters were selected: The tissue biopsies were sectioned at 7µm, fixed in 2% NBF (neutral buffered formalin) for 10min and stained with Hematoxylin for 7min. The pre-permeabilization step was performed with a 20min incubation at 37°C in 14U of collagenase type I (Life Technologies, Paisley, UK) diluted in 1xHBSS buffer (Thermo Fisher Scientific, Life Technologies, Paisley, UK) supplemented with 1% BSA (NEB, Ipswich, MA, USA). For permeabilization, the sections were incubated in 0.1% pepsin-HCl (pH 1) for 8min at 37°C. For the tissue removal, the tissue was first incubated for 1h at 56°C in beta-mercaptoethanol which was diluted in RLT buffer (x4).

After that, the tissue was treated with proteinase K diluted in PKD buffer at a ratio of 1:7 for 1h at 56°C, as previously described. The cDNA footprint was revealed by scanning the slide in an InnoScan 910 Microarray Scanner with 5µm resolution and gain at 10% or with a Metafer VSlide system at 20x resolution. An image showing the cDNA-footprint from one of the tissue optimizations can be seen in Fig. S2A-C.

Supplementary figures

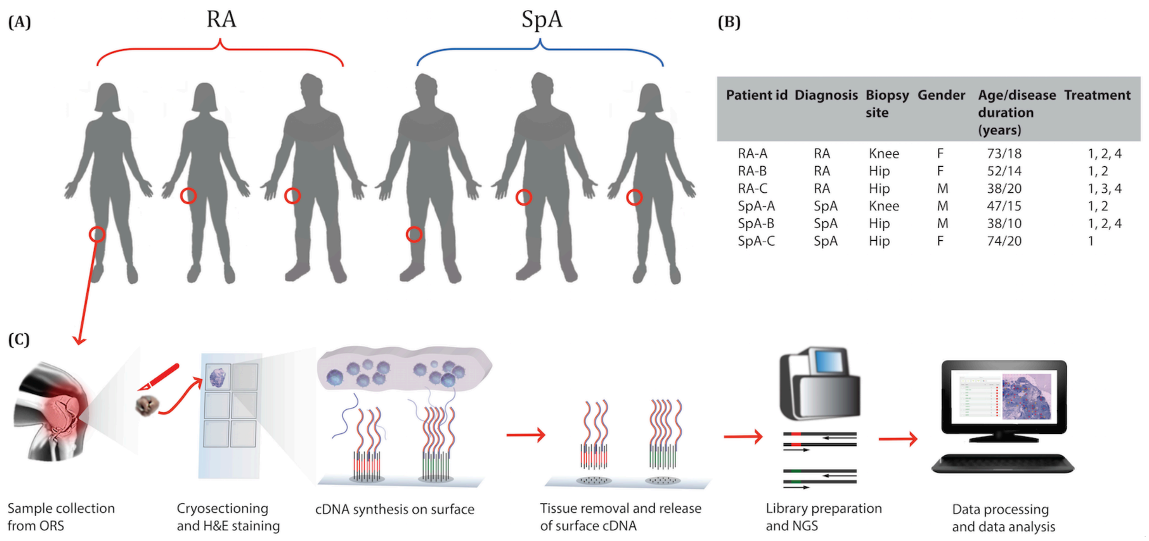

Patient id	Diagnosis	Biopsy site	Gender	Age/disease duration (years)	Treatment
RA-A	RA	Knee	F	73/18	1, 2, 4
RA-B	RA	Hip	F	52/14	1, 2
RA-C	RA	Hip	M	38/20	1, 3, 4
SpA-A	SpA	Knee	M	47/15	1, 2
SpA-B	SpA	Hip	M	38/10	1, 2, 4
SpA-C	SpA	Hip	F	74/20	1

Figure S1. Overview of the patient samples and the spatial transcriptomics assay
(A) Depiction of the joint biopsies (knee and hip) and brief patient characteristics. **(B)** Age, gender and disease duration at the timepoint of collection. Treatments at time of sampling include 1; DMARDs, 2; TNF inhibitors, 3; anti-IL-6R blockers, 4; glucocorticoids. **(C)** Depiction of the main steps of the spatial transcriptomics method starting with cryosectioning (three sections per biopsy), cDNA synthesis, tissue removal and release on surface cDNA, library preparation, DNA-sequencing and analysis.

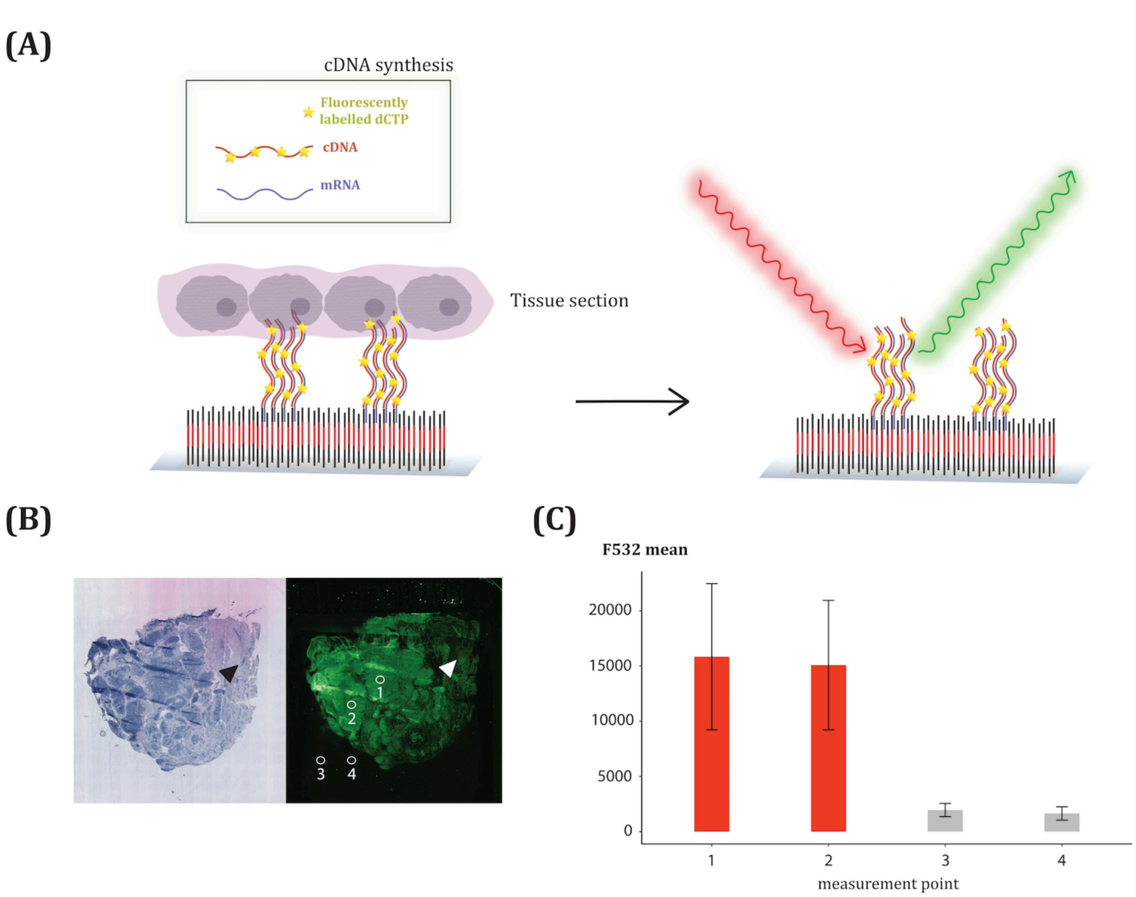

Figure S2. Spatial Transcriptomics optimization assay
(A) An overview of the cDNA synthesis step for the ST optimization assay. The surface probes are distributed all over the surface area, and cDNA synthesis is conducted with Cy3-labeled dCTPs in the nucleotide mixture and thereafter scanned with a 532 nm light source. **(B)** Shows the same section scanned with the Metafer VSlide system (H&E image to the left) and scanned on 532 nm after the tissue section was removed (to the right). The cDNA footprint can be seen with no signal diffusion and also less signal in areas with less cells (shown with an arrow). The small white circles 1-4 is areas in which the signal intensity was measured seen in **(C)** which shows the average signal intensities measured with GenePix Pro 5.0 from the circular areas with error bars.

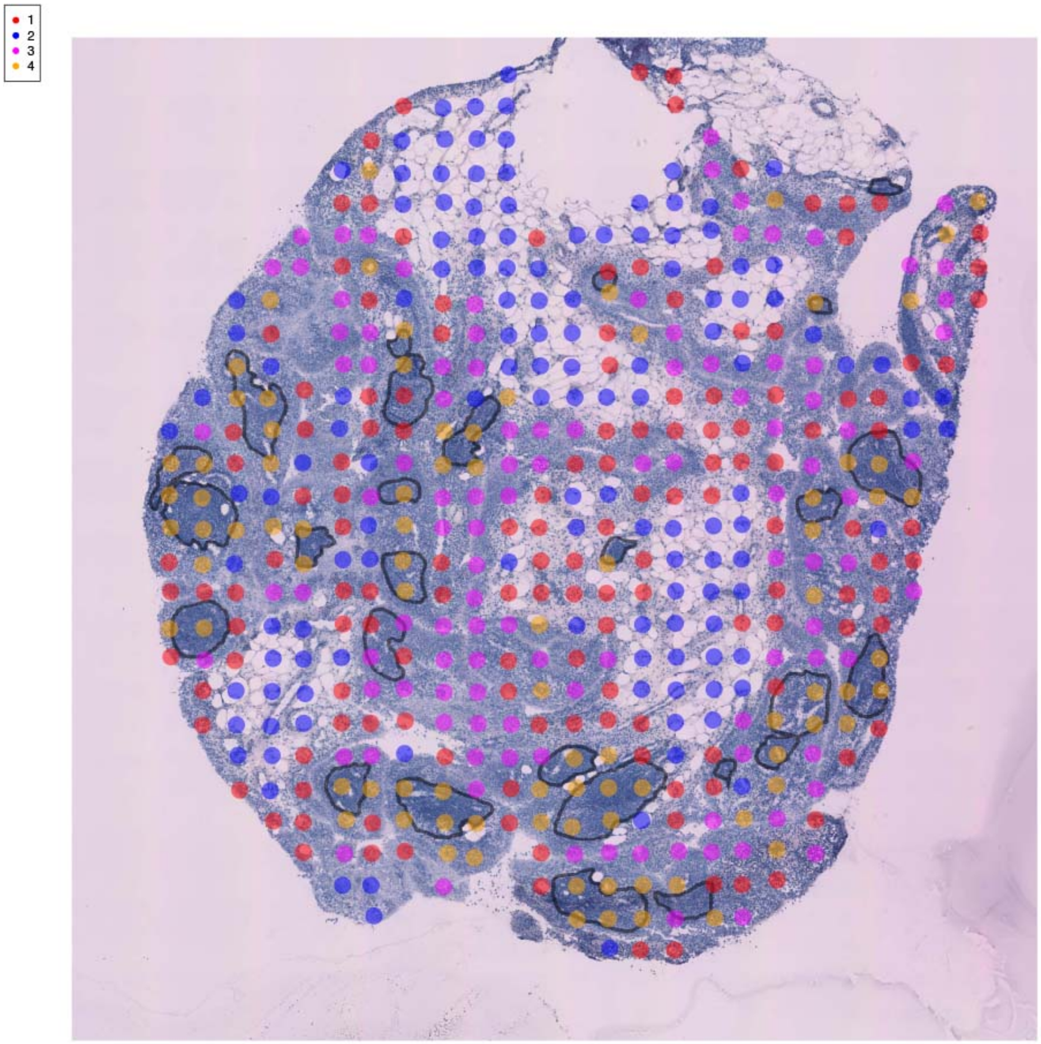

Figure S3. Cluster selection for RA-A 3
Black lines are regions annotated as lymphocyte infiltrates. Each spot represent areas in which mRNA was captured from. The numbers and colours represent the different clusters that were assigned. For the bulk analysis all spots were selected. For the infiltrate analysis cluster 4 was selected. For the outside-infiltrate analysis clusters 1 and 3 were selected.

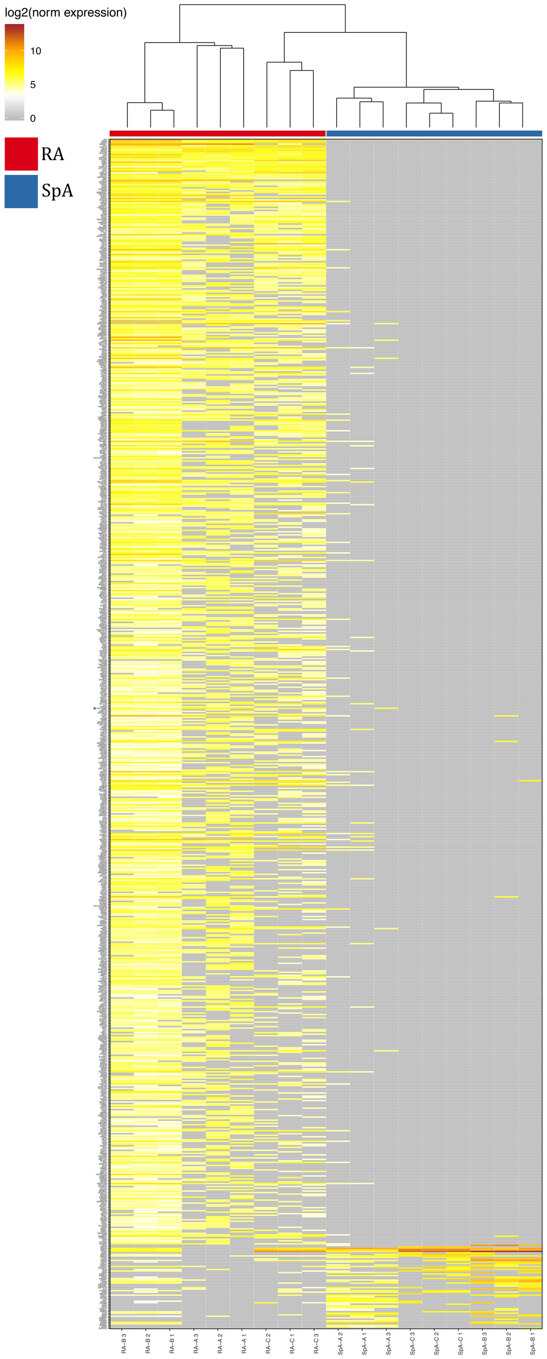

Figure S4. Larger set of genes from the DE analysis on the bulk data.
The dendrograms were ordered so that all RA-samples are to the left and all SpA-samples to the right. For each selection, all genes that had a log2fold change >2 and *p*-value lower than 0.01 from the DE-analysis are shown. N.B. the reference genome GRCh38 only maps to the haplotype HLA-DR1, which contains HLA-DRB1 and HLA-DRB5 which is a homolog of HLA-DRB4. It is possible that faulty mapping can occur for specific variable genes like the ones building the MHC structures. The genes known to have this issue have been marked with *.

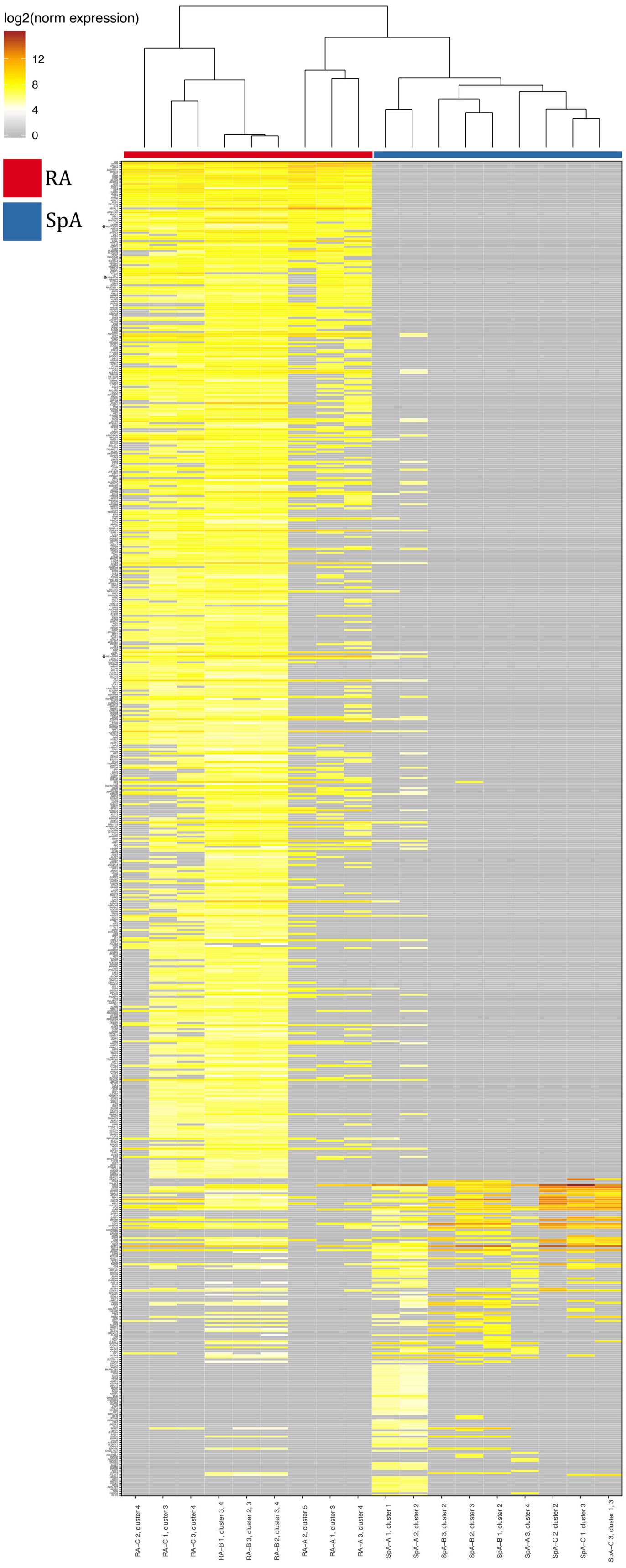

log2(norm expression)
12
8
4
0
RA
SpA
RA–C 2, cluster 4
RA–C 1, cluster 3
RA–C 3, cluster 4
RA–B 1, cluster 3, 4
RA–B 3, cluster 2, 3
RA–B 2, cluster 3, 4
RA–A 2, cluster 5
RA–A 1, cluster 3
RA–A 3, cluster 4
SpA–A 1, cluster 1
SpA–A 2, cluster 2
SpA–B 3, cluster 2
SpA–B 2, cluster 3
SpA–B 1, cluster 2
SpA–A 3, cluster 4
SpA–C 2, cluster 2
SpA–C 1, cluster 3
SpA–C 3, cluster 1, 3

Figure S5. Larger set of genes from the DE analysis of selected clusters correlating to infiltrate regions.
The dendrograms were ordered so that all RA-samples are to the left and all SpA-samples to the right. For each selection, all genes that had a log2fold change >2 and *p*-value lower than 0.01 from the DE-analysis are shown.N.B. the reference genome GRCh38 only maps to the haplotype HLA-DR1, which contains HLA-DRB1 and HLA-DRB5 which is a homolog of HLA-DRB4. It is possible that faulty mapping can occur for specific variable genes like the ones building the MHC structures. The genes known to have this issue have been marked with *.

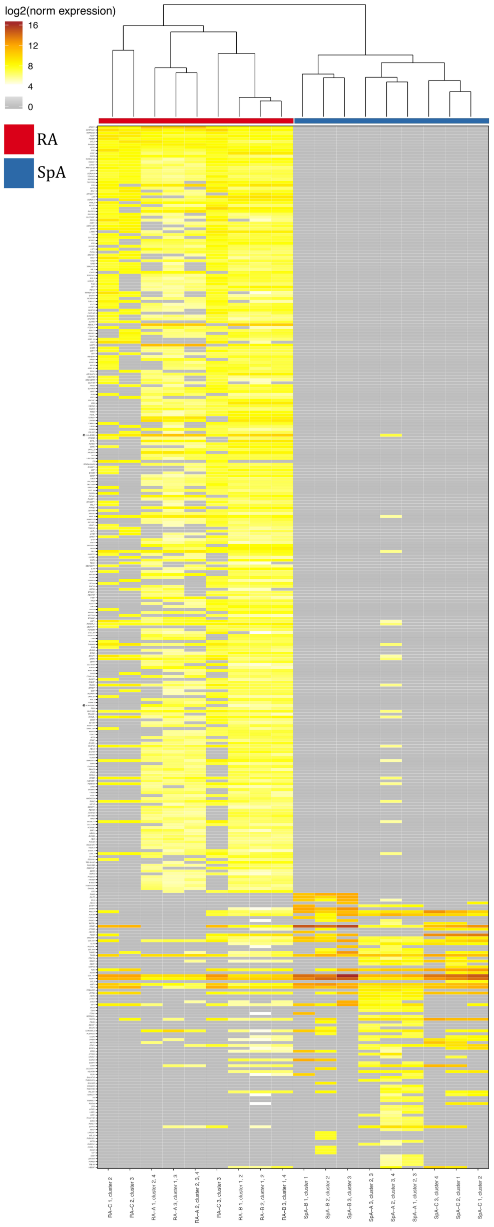

Figure S6. Larger set of genes from the DE analysis of selected clusters correlating to regions outside the infiltrates.

The dendrograms were ordered so that all RA-samples are to the left and all SpA-samples to the right. For each selection, all genes that had a log2fold change >2 and *p*-value lower than 0.01 from the DE-analysis are shown. N.B. the reference genome GRCh38 only maps to the haplotype HLA-DR1, which contains HLA-DRB1 and HLA-DRB5 which is a homolog of HLA-DRB4. It is possible that faulty mapping can occur for specific variable genes like the ones building the MHC structures. The genes known to have this issue have been marked with *.

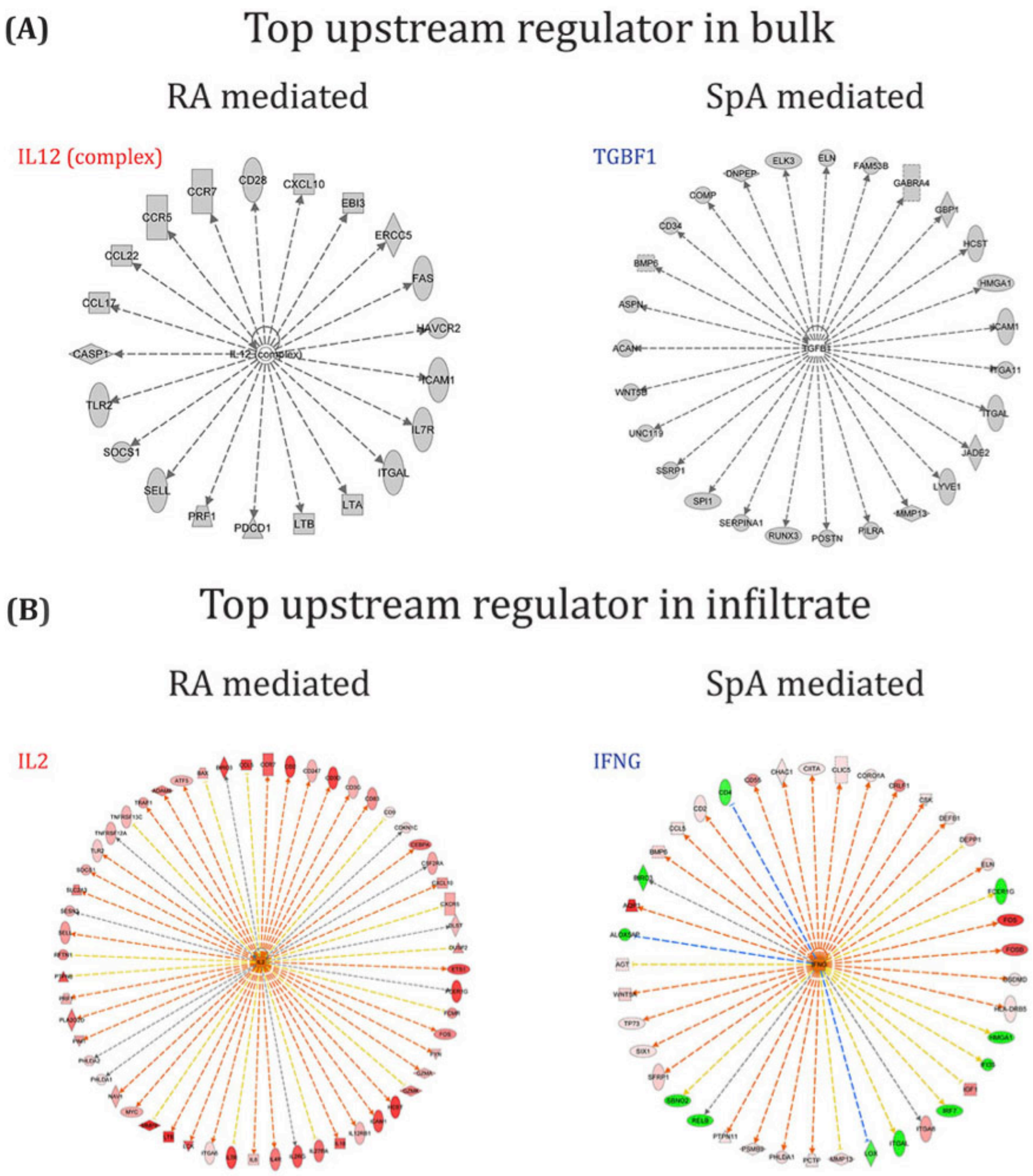

Figure S7. Top upstream regulators

Top upstream regulators for RA and SpA in the bulk RNA **(A)** and infiltrate areas **(B)**.

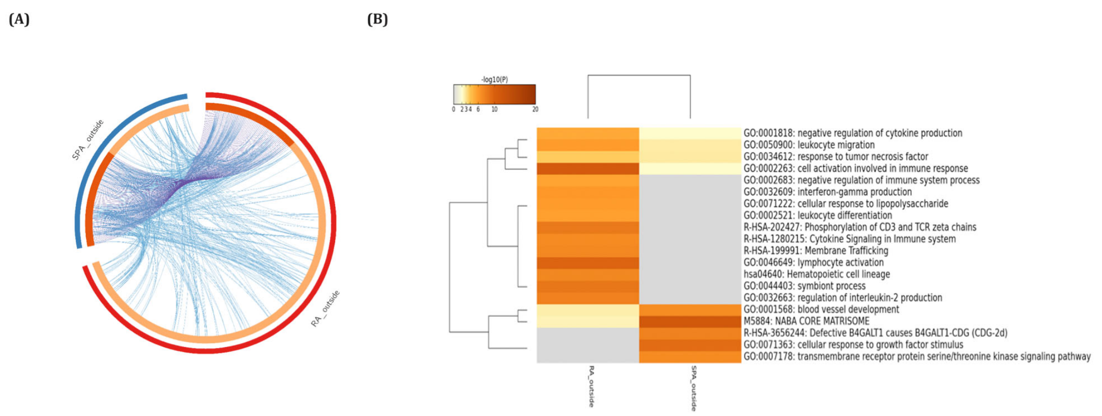

Figure S8. Circos plot and heatmap of enriched terms outside infiltrates
(A) The Circos plot shows how genes (hits – RA, 304 genes; hits 2 – SPA, 113 genes) from the input gene list overlap (please see description in Figure-2A legend). **(B)** Heatmap of enriched terms across input gene lists, coloured by p-values for the outside infiltrates analysis.

Supplementary tables

Table S1. Top canonical pathways from IPA on bulk data. In the tables, similarities are marked in blue.

RA (overlap %)	SPA (overlap %)
Primary Immunodeficiency signaling (20.5%)	Tumoricidal function of hepatic natural killer cells (12.5%)
Th1 pathway (11.6%)	Th1 and Th2 activation pathway (3.4%)
iCOS-iCOSL signaling in T helper cells (11.9%)	Th1 pathway (3.9%)
Regulation of IL-2 expression in activated and anergic T lymphocytes (13.9%)	Th2 pathway (3.4%)
CD28 signaling in T helper cells (11.0%)	Role of osteoblasts, osteoclasts and chondrocytes in RA (2.6%)
Th1 and Th2 activation pathway (9.5%)	Atherosclerosis signaling (3.2%)
TNFR2 signaling (20.7%)	Basal cell carcinoma signaling (4.3%)
T Cell Receptor Signaling (11.1%)	Calveolar-mediated endocytosis signaling (4.2%)
TWEAK Signaling (17.6%)	Glutathione biosynthesis (33.3%)
PKCθ Signaling in T lymphocytes (9.1%)	TWEAK signaling (5.9%)

Table S2. Top upstream regulators in the bulk data.

RA				SPA			
**Upstream regulators**	**Predicted activation state**	**p-value of overlap**	**Target molecules in dataset**	**Upstream regulators**	**Predicted activation state**	**p-value of overlap**	**Target molecules in dataset**
IL-12 (complex)	activated	1.37E-05	21	TGFB1		4.87E-06	27
IL-21	activated	1.41E-05	19	IFNG		9.52E-06	22
IL-2	activated	1.86E-05	41	MAP3K14		4.47E-05	5
SERPINE2		2.47E-05	8	SIRT1		4.74E-05	10

CD40LG	activated	3.02E-05	32	Alpha catenin		9.46E-05	6
FLT3LG		3.62E-05	10	VCAN		1.19E-04	6
DIO2		1.04E-04	7	NOTCH1		1.40E-04	8
ETS1	activated	1.18E-04	20	TP53		1.95E-04	22
MAP3K14	activated	1.20E-04	10	LY6E		2.46E-04	3
CSF2	activated	1.73E-04	33	SCX		2.46E-04	3

Table S3. Top canonical pathways from IPA on infiltrate data. In the tables, similarities are marked in blue. In the tables, similarities are marked in blue.

**RA (overlap %)**	**SPA (overlap %)**
CD28 signaling in T helper cells (16.5%)	Role of osteoblasts, osteoclasts and chondrocytes in RA (4.4%)
iCOS-iCOSL signaling in T helper cells (16.1%)	Axonal guidance signaling (2.9%)
Regulation of IL-2 expression in activated and anergic T lymphocytes (19.0%)	Virus entry via endocytic pathways (5.5%)
Th1 pathway (14.0%)	Leukocyte extravasation signaling (3.9%)
Primary immunodeficiency signaling (25.0%)	iCOS-iCOSL signaling in T helper cells (5.1%)
Th1 and Th2 activation pathway (11.7%)	CD28 signaling in T helper cells (4.7%)
T Cell Receptor Signaling (14.8%)	CDC42 signaling (4.7%)
PKCθ Signaling in T lymphocytes (12.3%)	CCR5 signaling in macrophages (5.4%)
Calcium-induced T lymphocyte apoptosis (20%)	Role of NFAT in regulation of the immune response (3.9%)
OX40 signaling pathway (20%)	Hepatic fibrosis/hepatic stellate cell activation (3.8%)

Table S4. Top upstream regulators within the infiltrate areas.

**RA**				**SPA**			
**Upstream regulators**	**Predicted activation state**	**p-value of overlap**	**Target molecules in dataset**	**Upstream regulators**	**Predicted activation state**	**p-value of overlap**	**Target molecule s in dataset**
IL-2	activated	1.14E-17	56	IFNG	activated	1.00E-10	41
IFNG	activated	1.88E-17	91	TGFB1	activated	3.16E-09	46
CD3	activated	9.92E-16	58	CHUK		1.92E-07	13
NFKB1	activated	1.12E-15	36	NOTCH1		3.58E-07	14
NFkB(complex)	activated	7.04E-15	55	IRS1		5.63E-07	10
CD40LG	activated	3.99E-14	42	GATA1		5.81E-07	13
IL-10		9.11E-14	39	IKBKG		6.57E-07	10
TNF	activated	1.99E-14	100	SIRT1		7.49E-07	16
RELA	activated	7.52E-13	41	TNF		1.19E-06	40
IL3	activated	1.07E-12	36	SFTPA1		1.73E-06	8

Table S5. Top canonical pathways from the outside infiltrate analysis. In the tables, similarities are marked in blue.

**RA (overlap %)**	**SPA (overlap %)**
Granulocyte adhesion and diapedesis (6.7%)	Role of osteoblasts, osteoclasts and chondrocytes in RA (3.9%)
Th2 pathway (6.9%)	Wnt/B-catenin signaling (3.6%)
Th1 and Th2 activation pathway (6.1%)	Role of macrophages, fibroblasts and endothelial cells in RA (2.3%)
Nur77 signaling in T lymphocytes (11.3%)	Differential regulation of cytokine production in intestinal epithelial cells by IL-17A and IL-17F (8.7%)
Role of NFAT in regulation of the immune response (5.9%)	Caveolar-mediated endocytosis signaling (4,2%)
Calcium- induced T Lymphocyte apoptosis (10%)	L-DOPA degradation (50%, ½)
Atherosclerosis signaling (6.5%)	Taurine Biosynthesis (50%, ½)
OX40 Signaling pathway (9.1%)	Granulocyte adhesion and diapedesis (2,4%)
Antigen presentation pathway (10.8%)	Thyronamine and iodothyronamine metabolism (33%,1 of 3)
CD28 signaling in T Helper cells (5.5%)	Thyroid Hormone metabolism I /33%,1 of 3)

Table S6. Top upstream regulators in the areas outside infiltrates

**RA**				**SPA**			
**Upstream regulators**	**Predicted activation state**	**p-value of overlap**	**Target molecules in dataset**	**Upstream regulators**	**Predicted activation state**	**p-value of overlap**	**Target molecules in dataset**
IFNG	activated	2.98E-14	17	TGFB1	activated	7.03E-15	39
IL4	activated	1.27E-09	17	PDGF BB		1.98E-09	14
FAS		2.22E-09	14	SCX		7.22E-08	5
TNF	activated	3.59E-09	19	WNT3A		1.46E-07	11
Ig		4.23E-09	16	SFTPA1		2.68E-07	7
STAT1	activated	5.04E-09	17	GREP1	activated	3.90E-07	6
MYC		6.11E-09	17	IFNG		4.47E-07	23
IRF7	activated	1.36E-08	14	EDN1	activated	1.64E-06	9
F2	activated	2.25E-08	17	IFNB1		1.69E-06	10
IL1B	activated	2.46E-08	16	TNF		1.77E-06	26
